# Effect of Cryogenic Treatment on Residual Stress and Microstructure of 6061 Aluminum Alloy and Optimization of Parameters

**DOI:** 10.3390/ma17194873

**Published:** 2024-10-04

**Authors:** Xuemei Niu, Zhi Chen, Linwang Jing, Yao Huang, Yuhang Liu

**Affiliations:** School of Mechanical Engineering, Taiyuan Science and Technology University, Taiyuan 030024, China; nxmlpc@126.com (X.N.); mechenzhi@tyust.edu.cn (Z.C.); jinglinwang1998@163.com (L.J.); hy343999539@163.com (Y.H.)

**Keywords:** cryogenic treatment, 6061 aluminum alloy, residual stress, microstructure evolution

## Abstract

Residual stress induced by solution treatment in 6061 aluminum alloy can lead to workpiece deformation, or even premature failure. The efficiency of traditional heat treatment for relieving residual stress is relatively low. Therefore, this study introduces a novel cryogenic treatment technique to reduce residual stress. The optimal cryogenic process parameters were achieved by orthogonal experiments: cryogenic temperature of 113 K, holding time of 24 h, 1 cryogenic cycle, and a cooling rate of 3 K·min^−1^, and the residual stress of aluminum alloy was measured by the blind hole method. The microstructural evolutions in 6061 aluminum alloy were tested by OM, SEM, and TEM. The results show that the introduction of cryogenic treatment can reduce residual stress in 6061 aluminum alloy by 64%, mainly due to the reduction of dislocations and the uniform distribution of β’’ phase.

## 1. Introduction

6061 aluminum alloy is widely used in transportation, aerospace, and other fields due to its excellent machinability, weldability, and formability [1,2,3]. In order to enhance the mechanical properties of aluminum alloy, heat treatment is typically applied after manufacturing. To increase the hardness and strength of the material, aluminum alloy parts undergo rapid quenching after solution treatment. However, rapid quenching causes severe temperature gradients within the material, inducing uneven plastic deformation. This will increase internal residual stress in aluminum alloy [4,5,6]. Numerous thin-walled aluminum alloy components are employed in the aerospace industry to reduce aircraft weight. Thin-walled components are characterized by low rigidity, and the release of residual stress can cause warping or deformation of thin-walled parts. Especially during machining, as material is removed, the internal stress balance of the alloy is severely disrupted, and the release of residual stress will significantly affect the dimensional stability of the product [7]. For example, the bushing components used in the aerospace field are commonly made from 6061 aluminum alloy. Due to their complex surface shapes and thin side walls, they are susceptible to deformation from residual stress, which affects dimensional stability [8]. In addition, excessive residual stress can even cause premature failure of the workpiece. Therefore, reducing the residual stress generated after solution treatment is crucial for ensuring the reliability of aluminum alloy components. 

There are many methods for eliminating residual stress. It can be classified based on their fundamental principles into heat treatment, mechanical treatment (such as vibratory stress relief, rolling, etc.), and surface heat treatment [9,10,11]. The mechanism for eliminating residual stress mainly involves applying force, heat, or magnetic fields. These methods introduce energy into the material, which releases the elastic strain energy or microplastic strain within it [12,13]. Heat treatment is the most widely used method for residual stress relief. This popularity is mainly due to its more mature technology and more stable effects. However, the effectiveness of traditional heat treatment in eliminating residual stress is limited. Younger et al. [14] conducted a thermal aging treatment to relieve the residual stress of aluminum alloy satellite box, and the results showed that the thermal aging treatment could reduce residual stress by 40%. Zheng, J.H. et al. [15] conducted a multi-step T74 aging treatment on AA7050 aluminum alloy, reducing the residual stress from 268 MPa to 196 MPa, which corresponds to a 27% reduction in residual stress. In order to increase the reduction rate of residual stress in cryogenic treatment, researchers investigated the effect of cryogenic treatment on residual stress 

Cryogenic treatment is often considered a supplement to traditional heat treatment. Cryogenic treatment can reduce residual stress without changing the shape and dimensions of the parts [16]. Liquid nitrogen is used as the coolant in cryogenic treatment, and the parts are held at a temperature range of 77–173K for a certain period during the process. Cryogenic treatment can refine grain size and promote the precipitation of phases, thereby reducing residual stress. Jingmin Li et al. [17] found that introducing cryogenic treatment between solution treatment and aging treatment can eliminate residual stress in aluminum alloys. Zeju Weng et al. [18] studied the effect of heat treatment sequences on residual stress in 7075 aluminum alloy. Their research showed that the solution-cryogenic-aging sequence most effectively reduces residual stress. The specimens treated with cryogenic treatment contained fine precipitates, and the reduction in residual stress improved dimensional stability. Diana A. Lados et al. [19] applied cryogenic-natural aging treatment to Al-Mg-Si alloys. The residual stress was significantly reduced, and a higher hardness was achieved because the internal material of alloy generated a stress field opposite to the quenching residual stress. Lijun Wei et al. [20] conducted cryogenic treatment on 7075 aluminum alloy with 0 °C, −60 °C, −120 °C, and −196 °C, and the specimen with a holding temperature of −120 °C had the lowest residual stress. Matic Jovičević-Klug et al. [21] pointed out that as the DCT holding time increased from 24 h to 48 h, the average size of precipitates in Al-Mg-Si alloys decreased and smaller precipitates enhanced the mechanical properties of the alloy. Yongxin Jia et al. [22] subjected T6-treated 2024 aluminum alloy to liquid nitrogen at −196 °C for various durations (0.5, 1, 2, 3, 4 h) and found that extending DCT time promoted the precipitation of the S phase. Gogte, C.L. et al. [23] applied CT at −185 °C to AA 6061 aluminum alloy for various time periods. The results show that hardness, electrical conductivity, and surface roughness all improved. 

Cryogenic treatment can improve the mechanical properties of alloys primarily because it can regulate the microstructure of the alloy, such as the grain size and the density of dislocations and secondary phases [24]. Fei Dong et al. [25] observed that Al–Cu–Mn alloys subjected to low-temperature plastic deformation exhibited significant grain refinement, a substantial reduction in the proportion of coarse secondary phases, and improvements in ultimate tensile strength, yield strength, and elongation. Ates, H. et al. [26] noted in cryogenic treatment experiments that dislocations tend to align along subgrain boundaries, which reduced internal energy, further refining grains and enhancing the tensile strength and elongation of the alloy. When Araghchi and M et al. [27] conducted a cryogenic treatment before an aging treatment, they found that the cryogenic treatment shortened the time to reach the aging peak, and the β phase (Mg_2_Si) content was extremely low, highly dispersed in the matrix, and reduced in size.

To eliminate the residual stress in 6061 aluminum alloy, numerous studies focused on optimizing traditional heat treatment techniques. However, there is little detailed discussion on introducing cryogenic treatment and analyzing the effects of different cryogenic parameters on residual stress. To address this gap, solution-aging and solution-cryogenic-aging treatment experiments were performed on the 6061 aluminum alloy. The orthogonal experiment and range analysis was employed to optimize the cryogenic treatment parameters. The alloy’s microstructure was characterized using optical microscopy (OM), scanning electron microscopy (SEM), and transmission electron microscopy (TEM). Ultimately, the hardness and mechanical properties of the alloy were measured. This study analyzed the effects of cryogenic treatment on residual stress, microstructure, and mechanical properties. It also discussed the mechanisms of cryogenic treatment for eliminating residual stress in aluminum alloys. The results show that cryogenic treatment can reduce residual stress by up to 64%. It also leads to a slight enhancement in mechanical properties. The main reason for the reduction of residual stress is the increased density and uniform distribution of precipitates β″ and a noticeable decrease in dislocation density. This research can provide some reference for the application of 6061 aluminum alloy in industries with high requirements for residual stress control.

## 2. Materials and Methods

The material selected in this study is 6061 T4 aluminum alloy. The chemical composition of the material is shown in Table 1. The sample materials were processed into cylindricals for measurement.

### 2.1. The Scheme of Heat Treatment

**Figure 1:**
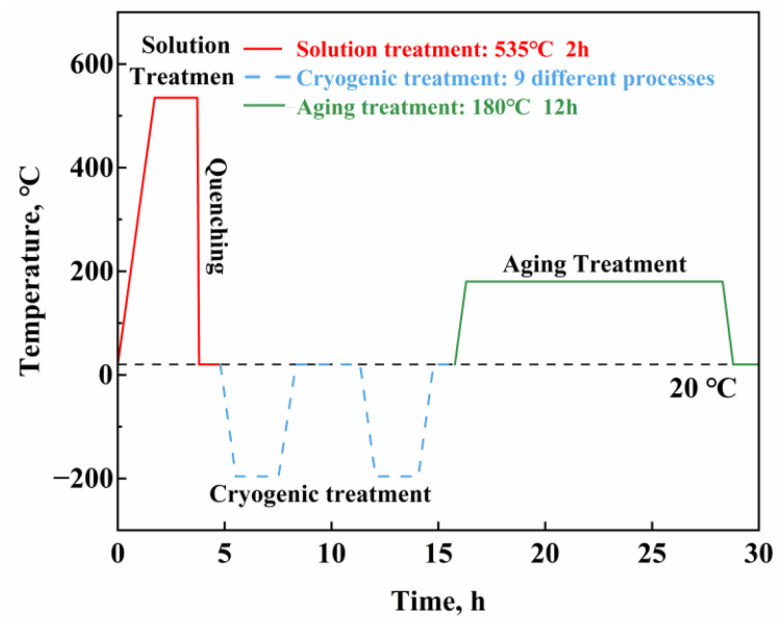
Heat treatment flowchart.

The specific heat treatment process is as follows: First, the aluminum alloy is treated by solid solution treatment at 535 °C for 2 h. After the solution treatment, the sample is quenched in water within 10 s. Then, the samples are divided into 10 groups. Sample 0 is used as a control group and does not undergo cryogenic treatment, and Samples 1 to 9 are treated using different cryogenic treatment. The cryogenic treatment is performed in a programmable SLX-30 cryogenic chamber. After cryogenic treatment, the samples are treated by aging treatment at 180 °C for 24 h in an HH-1S type CNC oil bath, with the aging time set to 9 h. The samples are air-cooled to room temperature. The specific heat treatment process is illustrated in Figure 1.

The effects of temperature, holding time, cryogenic cycles, and cooling rate on the residual stress of 6061 aluminum alloy are most pronounced. According to previous research [28,29], multiple cryogenic cycles can further promote the uniform distribution of precipitates, while the cooling rate refers to the rate of temperature change in the cryogenic chamber per unit time. This study designed two process schemes: solution treatment—cryogenic treatment—aging treatment and traditional solution treatment—axging treatment. The cryogenic treatment parameters were designed using a four-factor, three-level orthogonal experimental method, as shown in Table 2. Nine sets of orthogonal experimental parameters were obtained through orthogonal design, as shown in Table 3. The parameter selection is based on the work of other researchers [30].

In this study, the range analysis method is used to evaluate the impact of different experimental factors (such as temperature, holding time, cooling rate, and cryogenic cycles) on the residual stress results. First, the extreme value difference (the difference between the maximum and minimum extreme values) is calculated for each factor. The larger the difference, the greater the factor’s influence on the residual stress results. By ranking the extreme value difference, the importance of each cryogenic treatment factor in reducing residual stress can be determined. The formula for range analysis is as followed [31]:

(1) $$\bar{K_{xj}}=(r_{xj1}+r_{xj2}+r_{xj3})/3$$

(2) $$R_{0x\;}=\max(r_{xj1}+r_{xj2}+r_{xj3})$$

(3) $$R_{1x}=\max(r_{xj1}+r_{xj2}+r_{xj3})$$

(4) $$R_{x}=R_{x0}-R_{1x}$$

where $\bar{K_{xj}}$ represents the average of the residual stresses to factor *X* at the *j* level. $R_{x}$ represents the extreme value difference under the *X* factor, and it is the difference between the maximum and minimum residual stress values under the influence of *X* factor. $R_{x}$ reflects the range of variation in residual stress values under factor *X*. The larger $R_{x}$ indicate the greater impact of factor *X* on residual stress [32].

### 2.2. The Test Method of Residual Stress

**Figure 2:**
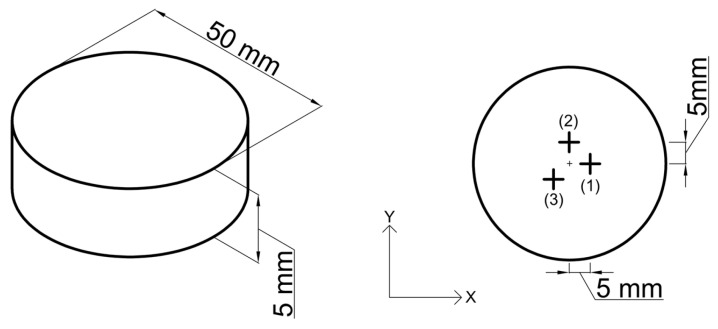
The schematic diagram shows the dimension of the sample and the test location of residual stress.

Cylindrical samples with a diameter of 50 mm and a thickness of 5 mm were used to measure residual stress. This study uses the blind hole method to measure the residual stress of samples treated by different processes. The strain was measured by BE(BA)120-2CD strain gages, and the test location was marked in the schematic diagram, as shown in Figure 2. The RS-200 drilling device, equipped with a high-speed air turbine-driven end mill with a rotational speed range of 20,000 to 400,000 rpm, was used to drill holes with a diameter of 1.803 mm and a depth of 1 mm in the test samples. Strain values were recorded using a D4 data acquisition system, and non-uniform stress was calculated using the integration method.

### 2.3. The Test Method of Microstructure

In order to conduct microstructure detection, the initial specimens were cut into cubes with a side length of 10 mm. The sample surfaces were polished sequentially with 400#, 600#, 800#, 1000#, 1200#, and 1500# abrasives, followed by mechanical polishing. The sample surface was etched with a 25% NaOH solution for 90 s. The microstructure was observed through a 4XC metallographic optical microscope (OM) and JSM-7900F scanning electron microscope (SEM). Moreover, the samples were polished to approximately 30 μm in thickness using sandpaper. The samples were then punched into Φ3 mm disks. Subsequently, thinning was performed using a Model 691 ion thinning system to prepare the samples for TEM (JEM-2100F) analysis.

### 2.4. The Test Method of Mechanical Properties

**Figure 3:**
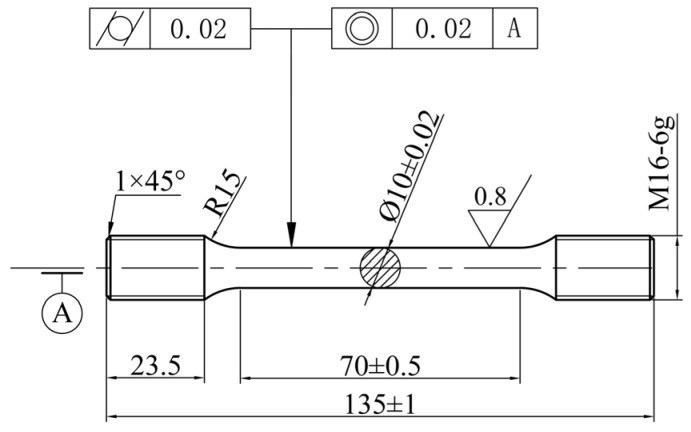
Diagram showing the dimension of tensile specimens.

Tensile tests were conducted using a SANS Zongheng Storm series universal testing machine, and samples were prepared according to the GB/T 228.1-2010 standard [33]. The dimensions of the tensile samples are shown in Figure 3. Before conducting the tensile tests, the sample surfaces were polished with sandpaper to ensure smoothness. The tensile test was conducted at a speed of 2 mm/min. Three tensile samples were prepared for each process, and the average value was taken as the final result. The microhardness of samples treated by different processes was measured by an HV-1000 microhardness tester with an error range of ±2%. The loading force for the test was 0.98 N, and duration times were 10 s.

## 3. Results and Discussion

### 3.1. Residual Stress

**Figure 4:**
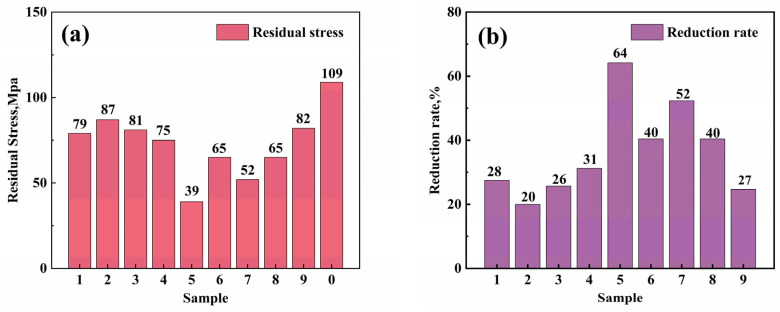
(**a**) Residual stress values of samples treated by different processes, and (**b**) residual stress elimination rates of samples treated by different processes.

Figure 4 shows the residual stress values on the surfaces of samples treated by different processes. The surface residual stresses after heat treatment were all compressive. Compared to the sample without cryogenic treatment (Sample 0), the residual stress of the samples treated with cryogenic treatment decreased. Sample 5 has the highest residual stress elimination rate. The residual stress value is 39 MPa, and the elimination rate reaches 64%.

To further analyze the effects of cryogenic treatment factors on residual stress, a range analysis of the experimental results was conducted. The residual stress results for each group of the orthogonal experiments are shown in Table 4. 

The results of the range analysis are shown in Table 5. The larger the range value, the greater its impact on residual stress. The ranking of importance can be seen from the table: Holding time > Cooling rate > Temperature > Cryogenic cycles. Range analysis can also determine the optimal parameters for cryogenic treatment. As shown in Table 4, the ranking of the average values in the temperature column is as follows: $\bar{K_{A3}}>\bar{K_{A1}}>\bar{K_{A2}}$. Holding time factor column: $\bar{K_{B2}}>\bar{K_{B1}}>\bar{K_{B3}}$. Cryogenic treatment cycles factor column:$\bar{K_{C2}}>\bar{K_{C3}}>\bar{K_{C1}}$. Cooling rate factor column: $\bar{K_{D1}}>\bar{K_{D3}}>\bar{K_{D2}}$. According to the range analysis results, the optimal cryogenic parameters to reduce residual stress in 6061 aluminum alloy are: cryogenic temperature of 113 K, holding time of 24 h, 1 cryogenic cycle, and a cooling rate of 3 K·min^−1^. The optimal process for reducing residual stress is the result of the combined effects of these four factors. For example, increasing the number of cryogenic cycles does not necessarily make the residual stress reduction higher; the minimum residual stress is achieved with just one cycle. As the results show, a cryogenic treatment can effectively eliminate the residual stress of 6061 aluminum alloy.

### 3.2. Microstructural Evolution

**Figure 5:**
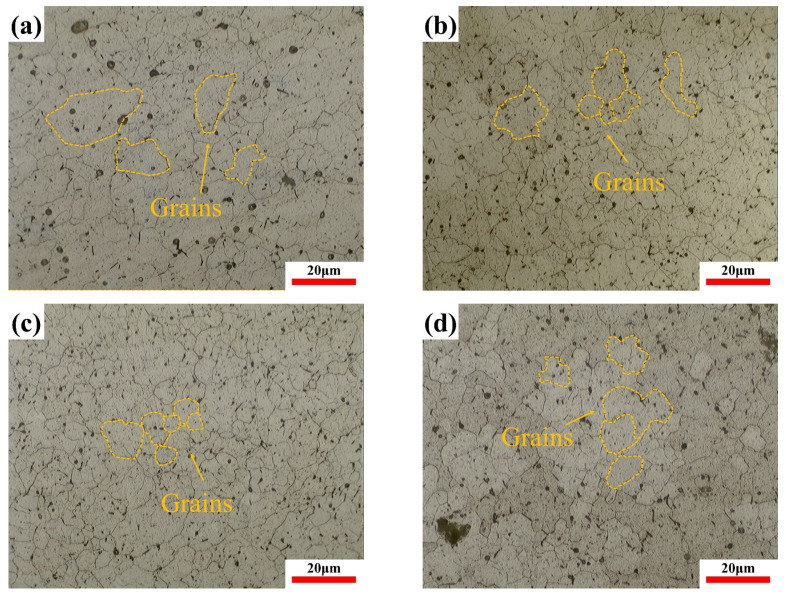
The OM images of samples treated by different processes: (**a**) sample 0; (**b**) sample 2; (**c**) sample 5; and (**d**) sample 7.

Figure 5 is the OM images of samples treated by different processes. In order to investigate the effect of cryogenic treatment on the grain size of 6061 aluminum alloys, OM images of the samples were imported into the Nano Measure software (Version 1.2) to measure the size of 50 grains. Each group was measured three times, and the average value was taken as the result. The results are shown in Table 6. The grain size of the sample without cryogenic treatment (Sample 0) was concentrated in the 5 to 15 nm range, which accounts for 83.7%, and grains in the 15 to 25 nm range accounted for 83.7%. The grain size of samples treated by cryogenic treatment was concentrated in the 0 to 10 nm range, which accounts for 85%, 92.5%, and 83.4%, respectively. Sample 5 has 45% of its grains concentrated in the 0 to 5 nm range.

According to previous studies [33], dislocation activity is restricted in small grains, and an increase in smaller grains helps weaken dislocation interactions. The uniform grain size leads to stress dispersion, reducing the accumulation of residual stress. The results indicate that after cryogenic treatment, the grain size becomes more uniform and the residual stress is relatively lower. It can be inferred that the reduction in grain size influences the residual stress in 6061 aluminum alloy by altering stress distribution and weakening dislocation movement.

**Figure 6:**
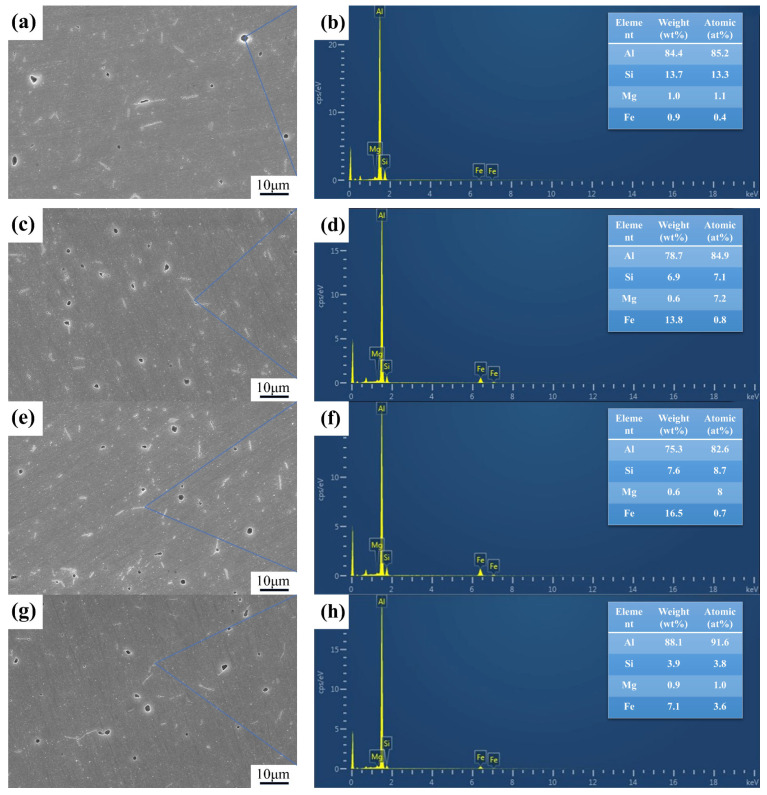
The SEM and EDS images of samples treated by different processes: (**a**,**b**) sample 0; (**c**,**d**) sample 2; (**e**,**f**) sample 5; and (**g**,**h**) sample 7.

The microstructure of 6061 aluminum alloy after heat treatment was tested by SEM, as shown in Figure 6. Black and white precipitates were observed on the surfaces of samples, and these precipitates are needle-like and rod-like in shape. As shown in Figure 6a, the precipitate phase in group 0 is sparse and unevenly distributed. This suggests that the uneven distribution of precipitates in the microstructure may affect the uneven distribution of residual stress. After cryogenic treatment, the number of precipitates within the alloy significantly increased, and the distribution of precipitates became more uniform, as shown in Figure 6c,e,g. To determine the composition of the precipitates, EDS analysis was performed. As seen in Figure 6b, the Si content is relatively high, while the Mg content is low. The atomic of ${\mathrm{Mg}}_{2}\mathrm{Si}$ is 2:1, which indicates that the black precipitate may be ${\mathrm{Mg}}_{2}\mathrm{Si}$ and Si-rich phase. In Figure 6d,f, there is a small amount of Fe atoms present, along with a higher Si content. These results suggest that the needle-like precipitates, ranging in size from 7 to 10 nm, may be a mixed phase of AlFeSi and Si-rich phases. For precipitates smaller than 5 nm, the Fe/Si atomic ratio is 0.95, as shown in Figure 6h. The Fe/Si atomic ratio for the β-AlFeSi phase is 0.8 to 1.3 [34]. Therefore, the smaller needle-like precipitates may be the β-AlFeSi phase. The β-AlFeSi phase has a monoclinic crystal structure and is hard and brittle. It can penetrate the matrix, leading to matrix cracking. The β-AlFeSi phase generally causes stress concentration at the needle tips, which promotes crack initiation and propagation, thereby reducing the tensile strength and plasticity of the alloy.

The low residual stress elimination rate of sample 0 is due to the few and unevenly distributed precipitates in the sample. The increase in precipitates after cryogenic treatment indicates that cryogenic treatment provides the driving force for the precipitation of the second phase. The residual stress elimination rate of sample 2 is higher because more precipitates are formed. The tensile stress field formed around the precipitates overlaps with the residual stress field after quenching, thus reducing the residual stress 0 [35]. The residual stress elimination rate of sample 5 reached 64%. It can be attributed to the dispersal distribution of precipitates in the alloy. The results indicate that increasing the number of precipitates and their dispersal distribution can reduce residual stress in 6061 aluminum alloys.

Precipitation strength is an important strengthening mechanism for 6061 aluminum alloys [36]. Cryogenic treatment promotes the precipitation and dispersion of strengthening phases. The increase of fine precipitates and the fragmentation of coarse precipitates can improve their pinning effect on dislocations, thereby reducing the residual stress within materials. The precipitation sequence of phases in 6061 aluminum alloy is as follows: supersaturated solid solution → GP zone (spherical) → β″ phase (needle-like) → β′ phase (rod-like) → β phase. The GP zone is coherent with the Al matrix, and the atoms at the interface are shared between the two phases. This results in lattice distortion at the boundaries, generating elastic forces that impede the movement of dislocations. The β″ phase is the primary strengthening phase in 6061 aluminum alloy, and its needle-like structure provides better strengthening effects compared to the rod-like β′ phase. This phase maintains coherence with the matrix along the <100> crystal planes, generating larger elastic distortions compared to the GP zone. These distortions impede dislocation movement, which forces dislocations to bypass the β″ phase. As the precipitation of the β phase increases, the obstruction to dislocations strengthens, and the phenomenon of dislocation accumulation decreases. This reduces stress concentration within the material, thereby reducing the residual stress field in alloy. Meanwhile, the increased obstruction to dislocation movement makes it more difficult for the material to undergo plastic deformation. This enhances the strength in 6061 aluminum alloy.

**Figure 7:**
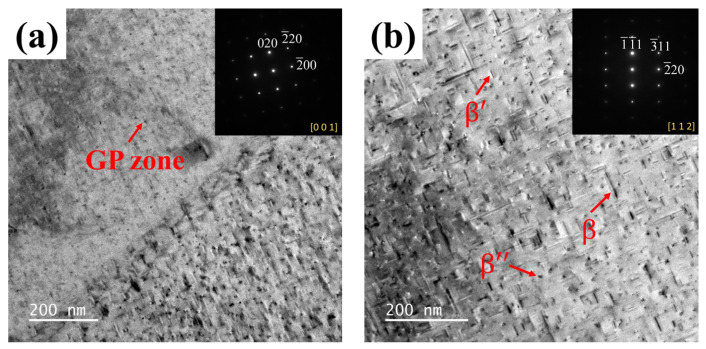
TEM images of specimens treated with different processes: (**a**) sample 0; and (**b**) sample 5.

In order to further investigate the microstructure of 6061 aluminum alloy, TEM was used to test samples treated by different processes. Figure 7a shows the TEM image of the sample without cryogenic treatment (Sample 0), and Figure 7b shows the TEM image of the treated by cryogenic treatment (Sample 5). In Figure 7a, the number of precipitates is relatively low, primarily appearing as dot-like and needle-like forms. In Figure 7b, it can be seen that after cryogenic treatment, the number of precipitates inside the sample increased significantly, not only with dot-like precipitates but also a large number of needle-like precipitates. SAED analyses of the precipitates confirm that the needle-like precipitates are ${\mathrm{Mg}}_{2}\mathrm{Si}$ [37]. The sizes of the precipitates indicated by the red arrows were measured as 67 nm, 17 nm, and 6 nm, respectively. According to previous research [38], these precipitates may be β, β’, and β″, respectively. The residual stress in Sample 5 is lower than that in Sample 0. This lower residual stress is because the cryogenic treatment causes a lattice contraction in alloy, which provides a strong driving force for precipitate formation, leading to the precipitation of solute atoms from the matrix [6]. Solute atoms tend to settle at defects, such as dislocations and grain boundaries [39]. The energy is higher at defects, and settling at these sites helps reduce the total internal energy of the alloy. During subsequent aging treatments, needle-like precipitates diffuse along dislocations and grain boundaries, allowing these precipitates to distribute uniformly throughout the alloy rather than accumulating in specific areas [40]. The precipitation of strengthening at defects also weakens the surrounding stress field, and this decreases the residual stress within the alloy. Therefore, cryogenic treatment reduces the residual stress and enhances material properties by promoting the precipitation of strengthening phases.

**Figure 8:**
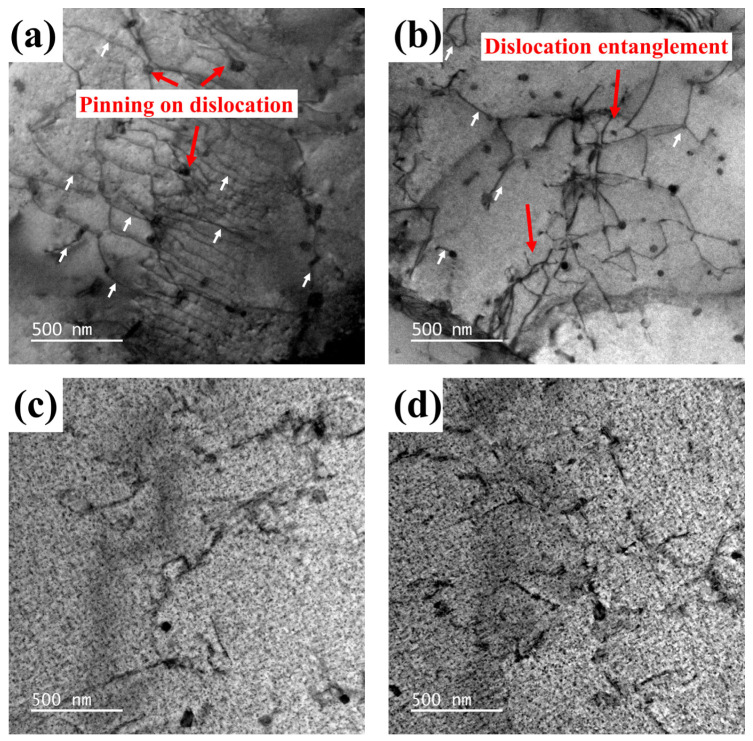
TEM images of specimens treated with different processes: (**a**,**b**) sample 0; and (**c**,**d**) sample 5.

The dislocation morphologies of the sample without cryogenic treatment (sample 0) and the sample treated by cryogenic treatment (sample 5) are shown in Figure 8. As shown in Figure 8a,b, the white arrows indicate the dislocation. A high density of dislocations is present in sample 0, with some dislocations obstructed by disk-shaped precipitates. The clear dislocation entanglement can be observed in sample 0. In contrast, Figure 8c,d shows that in sample 5, the density of precipitates is higher and dislocation distribution is more dispersed. This phenomenon can be attributed to the rapid temperature drop during the quenching of aluminum alloy, which results in different cooling rates in different regions of the alloy, leading to uneven thermal stress distribution. Such uneven thermal stress induces non-uniform plastic deformation, thereby increasing dislocation density [41]. A lower precipitate density will lead to dislocations easily bypassing the precipitates. Thereby, dislocation may gather in certain regions and interact with other dislocations.

Based on previous research [42], an increase in dislocation density will lead to a rise in local elastic strain energy, disrupting the internal stress balance of the alloy. As a result, samples subjected to solution treatment exhibit a strong residual stress field. However, cryogenic treatment can provide external energy to promote the precipitation of precipitates. Solute atoms tend to settle at defects to form precipitates, which will reduce the number of defects and the elastic strain energy within the alloy, achieving the goal of reducing residual stress. As shown in Figure 8b,c, fine β″ phases are densely precipitated within the grains. The smaller precipitates are more densely distributed in the matrix, with shorter average spacing between them. This makes it more difficult for dislocations to bypass these precipitates during movement, leading to a stronger pinning effect. This pinning effect hinders dislocation migration, resulting in a more dispersed dislocation distribution within the alloy, which helps to prevent localized stress concentration.

**Figure 9:**
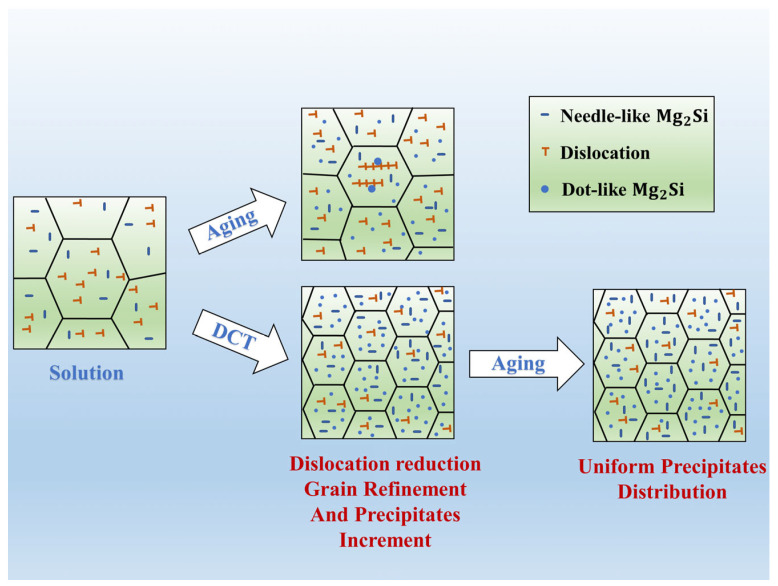
Grain refinement, dislocation movement and second phase precipitation mechanism.

The mechanisms of grain refinement, dislocation movement, and precipitation of secondary phases within aluminum alloys are used to explain the observed phenomena in the experiment, as illustrated in Figure 9. After the solution treatment of aluminum alloys, a high density of dislocations is present within the material. Typically, aging treatment is performed directly after solution treatment. But aging treatment does not impede dislocation migration. Due to the increased density of precipitates, the pinning effect on dislocations is enhanced, leading to severe dislocation accumulation, as shown in Figure 7a,b. This accumulation severely disrupts the internal stress balance of the material, resulting in high residual stress.

First, low temperature will lead to lattice contraction within the material when combined with cryogenic treatment. This contraction provides energy that facilitates dislocation movement and rearrangement, promoting dislocation annihilation [43]. Lattice contraction also provides a strong driving force for the enhanced precipitation of solute atoms, and the precipitation of strengthening phases consumes some of the elastic strain energy at defect, which reduces the residual stress within alloy. Second, at low temperatures, the significantly reduced atomic diffusion rate impacts grain growth. As the density of precipitates increases, they can pin at grain boundaries, inhibiting grain growth during subsequent aging processes and maintaining smaller grain sizes. Smaller grain sizes result in a more uniform stress distribution within the alloy. The increased number of grain boundaries obstructs stress transfer, leading to a more homogeneous stress distribution and a reduction in overall residual stress levels. Thereby, the sample 5 treated by cryogenic treatment shows a significant reduction in residual stress, and it can be attributed to the combined effects of refined grain size, the reduction of dislocation density, and increased fine precipitates.

### 3.3. Mechanical Properties

**Figure 10:**
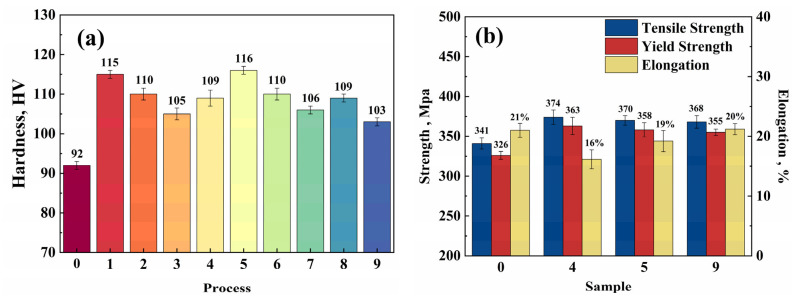
The results of samples with different treatments: (**a**) hardness; and (**b**) mechanical properties.

In most studies on reducing residual stress in aluminum alloys, the mechanical properties of the material after heat treatment are not tested. In practical applications, the service environments of materials are diverse. It is crucial not only to evaluate the effectiveness of residual stress elimination but also to ensure that eliminating residual stress does not excessively damage other properties of the component. This study included hardness testing and tensile testing to evaluate the mechanical properties of 6061 aluminum alloy, with results shown in Figure 7. The hardness of 6061 aluminum alloy after different treatments is illustrated in Figure 10a. The hardness of the sample without treatment (Sample 0) was 92 HV. After cryogenic treatment, the hardness of the samples increased by a range of 12–26%. The highest hardness was observed in Group 5, with a hardness of 116 HV. Therefore, a cryogenic treatment can enhance the hardness of 6061 aluminum alloy. In the low temperature environment, the precipitation of precipitates is accelerated, and this change can lead to an increased density of fine precipitates, as shown in Figure 7b.

The strength and elongation after different treatments of 6061 aluminum alloy are shown in Figure 10b. Compared to the untreated sample 0, the strengths of samples 4, 5, and 9 have improved, with increases in tensile strength of 9.7%, 8.5%, and 7.9%, respectively. The yield strength increases are 11.3%, 9.8%, and 8.9%, respectively. The improvement in the alloy’s yield strength is primarily related to the grain size, and grain boundary strengthening can be described by the Hall–Petch equation [44]:

$$\sigma_{y}=\sigma_{0}+\frac{k_{y}}{\sqrt{d}}$$

where $k_{y}$ and $\sigma_{0}$ are constants for aluminum alloy, and d is the average grain size. Strength $\sigma_{0}$ and grain size d exhibit a functional relationship. The Hall–Petch equation shows that the material’s strength is inversely proportional to the grain size: smaller grains lead to higher strength. According to the results, the grain size of the samples decreases and strength has improved after cryogenic treatment.

**Figure 11:**
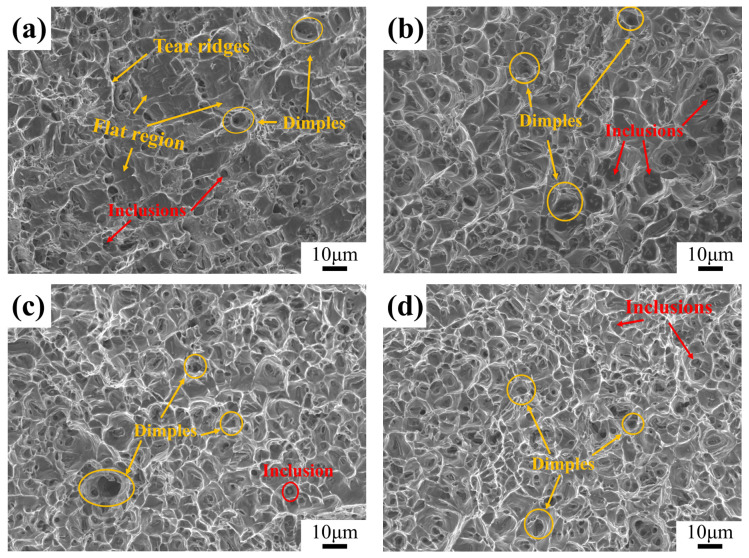
The fracture morphology of samples treated by different processes: (**a**) sample 0; (**b**) sample 2; (**c**) sample 5; and (**d**) sample 7.

In order to further investigate the tensile fracture morphology of 6061 aluminum alloy, an SEM analysis was conducted on the fracture surfaces, as shown in Figure 11. It can be seen from Figure 11 that the fracture surfaces of the tensile specimens all exhibit equiaxed dimples and tear ridges, indicating that all the samples have ductile fracture characteristics. Brittle fractures are often associated with flat regions, which are usually formed when cracks propagate along a specific direction during the fracture process. Flat regions can be observed in Figure 11a, suggesting that sample 0 has a brittle-ductile mixed fracture mode. In contrast, no obvious flat regions were found in the samples treated by cryogenic treatment, which are primarily characterized by numerous dimples of varying sizes and depths. This indicates that the fracture type of the samples treated by cryogenic treatment is ductile. Sample 2 has larger dimples, while samples 5 and 9 have numerous small dimples, as shown in Figure 11b–d. The dimples contain inclusions, which are secondary phase particles. The material properties between the secondary phase particles and the matrix are different, so these particles may act as initiation points for micro-cracks. The results show that the elongation of the samples treated by cryogenic treatment has decreased. Compared to sample 0, sample 2 shows a 5% elongation reduction, while samples 5 and 9 exhibit reductions of only 2% and 1%, respectively. Therefore, the hardness and strength of 6061 aluminum alloy are improved after cryogenic treatment, but the elongation after fracture decreases, with the lowest elongation reaching 16%. Although the elongation at break decreased slightly after cryogenic treatment, the reduction was minimal. Both the hardness and strength improved to a small extent, indicating that the key mechanical properties of the aluminum alloy were not significantly affected while residual stress was reduced.

## 4. Conclusions

This study provides a detailed investigation of the effects of cryogenic treatment on the residual stress and microstructure of 6061 aluminum alloys. The main conclusions are as follows:

(1) Combining cryogenic treatment with traditional heat treatment can significantly reduce the residual stress in 6061 aluminum alloys. The optimized cryogenic treatment parameters were obtained through orthogonal tests and range analysis: a cryogenic temperature of 113 K, a holding time of 24 h, 1 cryogenic cycle, and a cooling rate of 3 K·min^−1^, and the significance of process parameters on residual stresses reduction was as follows: Holding time > Cooling rate > Temperature > Cryogenic cycles.

(2) Cryogenic treatment refines the grain size and increases the amount of the second phase. According to EDS analysis, the needle-like precipitates are mainly AlFeSi phases, and the particles are mainly Mg_2_Si. 

(3) After cryogenic treatment, the reduction in dislocation density and the dispersed distribution of phase β″ are the main factors contributing to the reduction in residual stress, which also enhanced the hardness and mechanical properties of 6061 aluminum alloy.

(4) Cryogenic treatment can eliminate residual stress while ensuring good mechanical properties of aluminum alloys. The residual stress elimination rate reached 64%. The hardness, tensile strength, and yield strength all improved, while the elongation after fracture decreased, with the lowest being 16% in Sample 2. 

(5) The best overall mechanical properties among the cryogenic treatment samples are observed in the fifth group, with the cryogenic parameters as follows: a cryogenic temperature of 113 K, a holding time of 24 h, 1 cryogenic cycle, and a cooling rate of 3 K·min^−1^.

On one hand, research in the future could explore the application scenarios of cryogenic treatment in specific industries, such as designing the shape of the experimental samples directly simulating real components in order to evaluate their economic viability and benefits in practical applications. On the other hand, this study observed a decrease in elongation after cryogenic treatment. Subsequent work could explore combining cryogenic treatment with other post-processing techniques to mitigate its impact on elongation. This work will provide important references and guidance for the practical application of cryogenic treatment.

## Figures and Tables

**Table 1:** Chemical composition of the 6061 aluminum alloy.

Element	Si	Fe	Cu	Mg	Cr	Zn	Ti	Mn	Al
Wt-%	0.7	0.52	0.19	1.11	0.13	0.24	0.12	0.15	Bal.

**Table 2:** Parameter level definition.

Factors				
Level	Temperature/K (A)	Holding Time/h (B)	Cryogenic Cycles (C)	Cooling Rate/ K·min^−1^ (D)
1	153	2	1	1
2	113	12	2	3
3	77	24	3	5

**Table 3:** Orthogonal table.

Level	Temperature/K (A)	Holding Time/ h (B)	Cryogenic Cycles (C)	Cooling Rate/ K·min^−1^ (D)
0	-	-	-	-
1	153	2	1	1
2	113	12	2	1
3	77	24	3	1
4	153	12	3	3
5	113	24	1	3
6	77	2	2	3
7	153	24	2	5
8	113	2	3	5
9	77	12	1	5

“-” means without cryogenic treatment.

**Table 4:** The results of residual stress for each group.

Cryogenic Treatment					
Level	Temperature/K (A)	Holding Time/ h (B)	Cryogenic Cycles (C)	Cooling Rate/ K·min^−1^ (D)	Residual Stress
0	-	-	-	-	109
1	153	2	1	1	79
2	113	12	2	1	87
3	77	24	3	1	81
4	153	12	3	3	75
5	113	24	1	3	39
6	77	2	2	3	65
7	153	24	2	5	52
8	113	2	3	5	65
9	77	12	1	5	82

**Table 5:** The results of range analysis.

Factors				
Level	Temperature/K (A)	Holding Time/ h (B)	Cryogenic Cycles (C)	Cooling Rate/ K·min^−1^ (D)
k1	69	80	67	82
k2	64	81	72	60
k3	76	57	69	71
R	12	24	5	22
Influence degree	Holding time > Cooling rate > Temperature > Cryogenic cycles			
Optimal combination	113-24-1-3			

**Table 6:** The results of grain size distribution.

Number	0–5 nm	5–10 nm	10–15 nm	15–20 nm	20–25 nm
Sample 0	9.3%	62.8%	20.9%	2.3%	4.7%
Sample 2	20.0%	65.0%	12.5%	0	2.5%
Sample 5	45%	47.5%	7.5%	0	0
Sample 7	22.0%	63.4%	12.2%	2.4%	0

## Data Availability

All data that support the findings of this study are included within the article.
